# Noninvasive serum *N*-glycans associated with ovarian cancer diagnosis and precancerous lesion prediction

**DOI:** 10.1186/s13048-024-01350-2

**Published:** 2024-01-27

**Authors:** Si Liu, Chang Tu, Haobo Zhang, Hanhui Huang, Yuanyuan Liu, Yi Wang, Liming Cheng, Bi-Feng Liu, Kang Ning, Xin Liu

**Affiliations:** 1 The Key Laboratory for Biomedical Photonics of MOE at Wuhan National Laboratory for Optoelectronics-Hubei Bioinformatics & Molecular Imaging Key Laboratory, Systems Biology Theme, Department of Biomedical Engineering, College of Life Science and Technology, Huazhong University of Science and Technology, Wuhan, 430074 China; 2 Department of Epidemiology and Health Statistics, School of Public Health, Fujian Medical University, Fuzhou, China; 3 Department of Laboratory Medicine, Tongji Hospital, Tongji Medical College, Huazhong University of Science and Technology, Wuhan, China

**Keywords:** Ovarian cancer, Serum, Orthogonal strategies, *N*-glycome, Early diagnosis

## Abstract

**Background:**

Ovarian cancer (OC) is one of the most common gynecological tumors with high morbidity and mortality. Altered serum *N*-glycome has been observed in many diseases, while the association between serum protein *N*-glycosylation and OC progression remains unclear, particularly for the onset of carcinogenesis from benign neoplasms to cancer.

**Methods:**

Herein, a mass spectrometry based high-throughput technique was applied to characterize serum *N*-glycome profile in individuals with healthy controls, benign neoplasms and different stages of OC. To elucidate the alterations of glycan features in OC progression, an orthogonal strategy with lectin-based ELISA was performed.

**Results:**

It was observed that the initiation and development of OC was associated with increased high-mannosylationand agalactosylation, concurrently with decreased total sialylation of serum, each of which gained at least moderately accurate merits. The most important individual *N*-glycans in each glycan group was H7N2, H3N5 and H5N4S2F1, respectively. Notably, serum *N*-glycome could be used to accurately discriminate OC patients from benign cohorts, with a comparable or even higher diagnostic score compared to CA125 and HE4. Furthermore, bioinformatics analysis based discriminative model verified the diagnostic performance of serum *N*-glycome for OC in two independent sets.

**Conclusions:**

These findings demonstrated the great potential of serum *N*-glycome for OC diagnosis and precancerous lesion prediction, paving a new way for OC screening and monitoring.

**Supplementary Information:**

The online version contains supplementary material available at 10.1186/s13048-024-01350-2.

## Introduction

Ovarian cancer (OC) is the leading cause of death in gynecological cancers with high morbidity and mortality [1, 2]. OC originates from benign neoplasms that share several features with ovarian tumors, such as ovarian cysts, thus it is significant if the precancerous lesion could be predicted. Early detection or screening could significantly improve the survival rate, and reduce the patient sufferings or economic losses [3]. However, many patients are diagnosed at late stages of cancer due to vague and late-occurring symptoms, as well as a lack of effective early diagnostic markers. CA125 [4] and HE4 [5] are the routine biomarkers for OC diagnosis, but they usually suffer from low sensitivity and specificity. Therefore, in-depth exploration of the unique molecular fingerprints for cancer stratification is of significance for personalized therapeutic management and improved patient care.

It has been reported that glycans are directly involved in a large number of diseases [6–8]. Glycoscience is necessary to achieve precision medicine goals, and research in this area will help leverage the strengths of genomics and proteomics to drive research transformation [6]. Glycosylation is one of the most common posttranslational modifications (PTMs) in mammals [9], which governs physiology and contributes to disease due to its participation in many key biological processes, such as cell adhesion, molecular trafficking and clearance [10]. Protein glycosylation plays an integral role in tumor immunosurveillance and immunotherapies [11, 12]. Significantly, serum-based noninvasive markers are more accessible than tumor tissues or cells for disease screening and in vitro detection [13]. In recent decades, serum protein N-glycosylation has been extensively explored in many cancers, suggesting that specific glyco-markers are candidates for cancer diagnosis and prognosis [14]. In terms of ovarian cancer, it was found that particular serum *N*-glycans featuring high-mannose (Man5, Man6, Man7 and Man8) or core-fucose (H6N5S3F1, H6N5S3F2, H7N6S3F1, H7N6S3F2, H7N6S4F1, H7N6S4F2 and H7N6S4F2) were potential candidate biomarkers for OC diagnosis and showed higher diagnostic performance than the routine tumor marker CA12-5 [15]. In addition, specific *N*-glycans (H3N5, H6N3, H4N5 and H5N5S2F1) in serum were capable of distinguishing controls from both pre- and post-treatment cancer patients [16]. Moreover, alterations in serum *N*-glycome (H4N4, H3N5, H4N3S1, H4N4S1, H5N5S1F2, H6N4S2F1 and H6N5S1F2) could predict primary resistance to standard chemotherapy [17]. The glycoprofiles of *N*-glycans (H5N4F1, H5N4S2F1 and H6N5S2) from abundant glycoproteins in serum could discriminate between different stages of ovarian cancer and healthy controls [18]. However, it has not been investigated whether serum *N*-glycome profile is differentially significant in the development of ovarian precancerous lesions. Thus, the exploration of serum *N*-glycome could improve our understanding of the tumor microenvironment and provide new avenues for clinical treatment [19].

In this study, we exploited a high-throughput assay to characterize the serum *N*-glycome profile in healthy controls and ovarian disease patients including benign neoplasms and different stages of ovarian cancer. The profiles of serum *N*-glycans were quantitatively compared between healthy controls and ovarian disease patients. The diagnostic performance of *N*-glycans was assessed by multiple parameter analysis. Additionally, the correlation between two routine biomarkers for OC diagnosis and serum protein N-glycosylation was evaluated. Consequently, a machine learning based discrimination model was employed to further elucidate the performance of serum *N*-glycome for early detection of OC.

## Materials and methods

### Study populations

The cohorts in this study consisted of healthy controls, benign neoplasms, and cancer patients with primary stages and advanced stages (Table S1). Ovarian disease patients consisted of benign neoplasms (*N* = 70) and cancer patients (*N* = 70) containing four stages of tumor node metastasis (TNM), the age in both of which were matched. On the contrary, the levels of CA125 and HE4 were significantly different between benign and cancer groups. Individuals with benign neoplasms were diagnosed with ovarian cysts or serous adenomas. In the cancer groups, the primary stages were stage I-II, and the advanced stages were stage III-IV. It should be noted that almost all the cancer patients have high-grade serous carcinoma (HGSC). A cohort of healthy blood samples (*N* = 60) was enrolled. All samples were collected from Tongji Medical College of Huazhong University of Science and Technology. The study was performed in accordance with the principles of the Declaration of Helsinki criteria, and was approved by the Ethics Committee of Tongji Hospital (TJ-IRB20221109), Tongji Medical College of Huazhong University of Science and Technology.

### *N*-glycan release and derivatization

*N*-glycans from human serum were released by PNGaseF digestion using Protein Deglycosylation Mix II (New England Biolabs, Ipswich, MA, USA), followed by separation and purification through solid-phase extraction (SPE) [20]. Briefly, 10 µL of serum was dissolved in ultra-pure water followed by the addition of 3.6 µL of PNGaseF buffer and 2.4 µL of denaturing buffer, giving a total volume of 100 µL. After denaturation in a Thermo-Shaker (Ningbo Hinotek Technology Co., Ltd) and cooling to room temperature, 12 µL of NP-40 and 5 units of PNGaseF were sequentially added. The mixtures were incubated at 37 ℃ overnight, and then the glycans were captured by solid phase extraction (SPE) with porous graphitized carbon (PGC, Sigma-Aldrich, St. Louis, MO, USA), and further concentrated under vacuum concentrator (Eppendorf, Germany). To avoid the loss of sialic acid during MALDI-MS detection, methylamidation of carboxyl group in the glycans was conducted according to our previous study [21]. Briefly, the sample was dissolved in 25 µL dimethyl sulfoxide (DMSO, Sigma-Aldrich, St. Louis, MO, USA) containing 1 M methylamine hydrochloride and 0.5 M N-methylmorpholine. Subsequently, 25 µL DMSO containing 50 mM (7-azabenzotriazol-1-yloxy) trispyrrolidinophosphonium hexa-fluorophosphate (PyAOP, Sigma-Aldrich, St. Louis, MO, USA) was added. The mixture was incubated at ambient temperature for 30 min, then the derivatized glycans were purified through SPE with microcrystalline cellulose (MCC, Sigma-Aldrich, St. Louis, MO, USA) [22]. The column with MCC as solid phase was initially washed with 3 mL of acetonitrile (ACN, Merck KGaA, Darmstadt, Germany), followed by the addition of 3 mL of equilibration solution (butanol: ethanol: water = 4:1:1). After loading the samples dissolved in equilibration solution, the derivatized *N*-glycans were eluted using 1mL of 50% of ethanol (Merck KGaA, Darmstadt, Germany).

### MALDI-MS analysis

After the serum glycoprotein *N*-glycans were purified by SPE, the glycome spectrum was analyzed by autoflex maX MALDI-TOF-MS (Bruker Daltonics, Germany). Briefly, the dried sample was dissolved in 5 µL 50% ACN, 0.5 µL of which was added to the MALDI plate. After the air drying, the equal amount of 10 mg/mL 2,5-Dihydroxybenzoic acid (DHB, Sigma-Aldrich, St. Louis, MO, USA) containing 50 mM sodium acetate (Sigma-Aldrich, St. Louis, MO, USA) was added. The *m/z* range was set at 1000–4500, and a total of 1000 laser shots were applied to each sample spot. MS data were acquired in the positive ion reflector mode. All samples were randomly spotted onto the plate in triplicate to overcome the batch effect. Glycan compositions were assigned with the aid of GlycoMod (http://web.expasy.org/glycomod/), and the *N*-glycan structures have also been confirmed by nanoLC-PGC-MS/MS analysis [23]. Briefly, aqueous glycan solutions were injected into a NanoLC Ultrasystem equipped with a PGC enrichment column and PGC analytical column, and separated with a 58-minute gradient of solvent A (5% ACN containing 0.1% FA) and solvent B (95% ACN containing 0.15 FA). Glycans were eluted from the PGC column with a linear gradient, as follows: 0–5 min, 95% A; 5-20 min, 95–92.5%; 20-26 min, 92.5–81.55% A; 26-42 min, 81.5–5% A; 42-46 min, 5% A; 46-52 min, 5–95% A; 52-58 min, 95% A. MS spectra were acquired in positive mode with a mass range of 500–4000 with an acquisition time of 0.3 s per spectrum. MS/MS spectra were acquired by different collision energies over an *m/z* range of 50-2000 with acquisition time of 0.1s per spectrum. Additionally, the serum *N*-glycome profile was consistent with previous literature [24–27]. GlycoWorkBench 2.1 software was used to visualize the glycan structures.

### Lectin-ELISA

The concentration of serum protein N-glycosylation was evaluated by OD values using ELISA kits (Boerfu Biotechnology), including ConA, SNA, MAL-II and PHA-E. Briefly, serum samples were diluted 5-fold with dilution buffer, followed by the addition to each well of a 96-well plate. After 1.5 h incubation at 37 ℃, 100 µL of biotinylated antibodies were added and incubated for 1 h. The plate was washed three times using washing buffer, and then HRP-conjugated streptavidin was added followed by the addition of TMB working solution and stop solution. Immediately, the absorbance was measured at 450 nm.

### Data processing and statistical analysis

The MALDI-MS data were exported as text files, which were further processed by MassyTools software (version 0.1.6.3) [28]. The percentage of each *N*-glycan was obtained by normalization in all serum *N*-glycans identified in this study. Quality control has been evaluated as described in our previous study [23], showing the repeatability and robustness of our method for *N*-glycome analysis by MALDI-MS. Considering the important role of several glycan features in disease progression, further analysis of glycan derived traits would inform our understanding of *N*-glycosylation mediated regulation in OC. Thus, the percentages of *N*-glycosylation were calculated by grouping the same glycan structures as described previously [23], including fucosylation, galactosylation, sialylation and bisection. Initially, the distribution of *N*-glycans was assessed by Kolmogorov-Smirnov (K-S) test. One-way analysis of variance (ANOVA) was performed in the comparison between three groups when the residues conformed to a Gaussian distribution, and multiple corrections were tested by Bonferroni, to control the false discovery rate (FDR) in many comparisons. In contrast, Kruskal-Walli’s test was performed to test the non-Gaussian distribution, and the post hoc test was conducted by Dunn’s test. Unsupervised principal component analysis (PCA) was conducted to assess the classification between controls and patients with benign or cancer, and linear discriminant analysis (LDA) was performed to evaluate the discriminative power of serum *N*-glycome in precancerous lesions. The diagnostic performance of serum protein *N*-glycosylation was evaluated by receiver operating characteristic (ROC) curve. ANOVA, ROC analysis and Pearson correlation were performed using GraphPad Prism 8, and PCA was produced by R. *P*-value less than 0.05 was considered statistically significant.

### Bioinformatics analysis

To make the diagnostic model mentioned above more acceptable, the individuals in this study were split into discovery and validation sets according to the time when samples were collected (Table S2). Random forest (RF) was used to build the three-classification model for the diagnosis of OC patients. RF was implemented by R 4.2.1 with *ranger* package (version 0.14.1), using the default parameter of ntree = 500. To elucidate the accuracy of the result, 5-fold cross validation was subsequently conducted within R. The performance of the model was assessed by several commonly used multi-classification evaluation indicators: binary AUC for each category (in this case, NormalAUC, BenignAUC and CancerAUC), MacroAUC and MicroAUC [29]. NormalAUC: Calculate metrics for label normal (controls) and label non-normal (including benign neoplasms and cancer patients). NormalAUC could be used to measure the model’s ability to distinguish controls and other samples including benign neoplasms and cancer patients. BenignAUC: Calculate metrics for label benign (benign neoplasms) and label non-benign (including controls and cancer patients). BenignAUC could be used to measure the model’s ability to distinguish benign neoplasms and other samples including controls and cancer patients. CancerAUC: Calculate metrics for label cancer (cancer patients) and label non-cancer (including controls and benign neoplasms). CancerAUC could be used to measure the model’s ability to distinguish cancer patients and other samples including controls and benign neoplasms. Macro-averaging refers to the arithmetic average of each statistical index value in all categories, whereas Micro-averaging averages the corresponding elements of each confusion matrix to obtain TP, FP, TN, and FN and then calculates the corresponding average. MacroAUC: Calculate metrics for each label (normal, benign and cancer), and find their unweighted mean. MicroAUC: Calculate metrics globally by considering each element of the label indicator matrix as a label. Both MacroAUC and MicroAUC could be used to measure the model’s global ability to distinguish controls, benign neoplasms and cancer patients. MicroAUC is susceptible to influence of the label imbalance, whereas MacroAUC treats all labels equally. The optimal number of features used for the discriminative model was examined through twenty-repeats 5-fold cross validation, resulting in the importance of features for the classification.

## Results

### Serum protein N-glycosylation in the development of OC

**Fig. 1:**
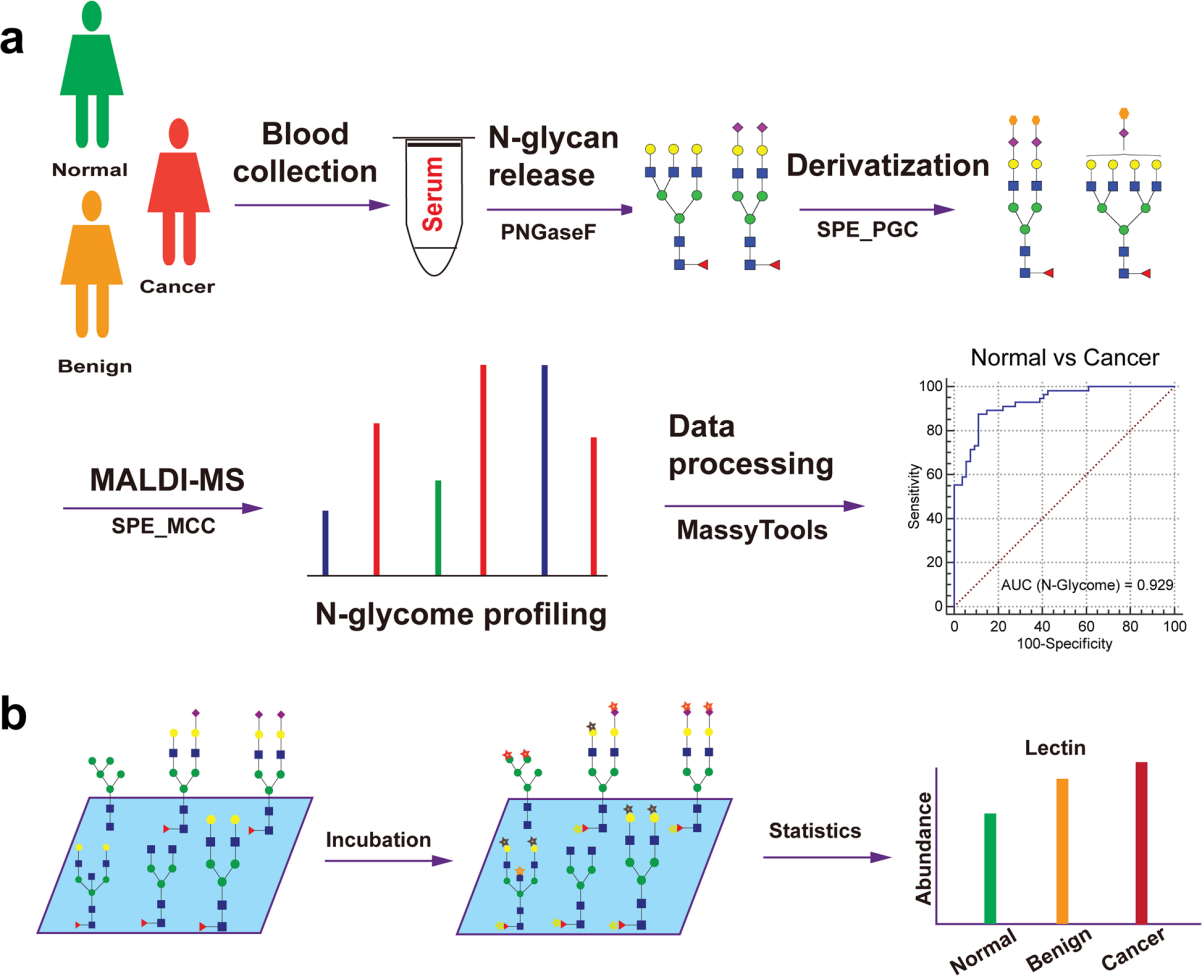
Schematic workflow of serum *N*-glycome profiling by orthogonal high-throughput strategy. **a** Serum *N*-glycome in ovarian cancer was characterized by MALDI-MS; (**b**) Differential expression of specific glycosylation was evaluated by lectin-based ELISA. Blue square denotes N-acetylglucosamine, green circle denotes mannose, yellow circle denotes galactose, purple diamond denotes N-acetylneuraminic acid and red triangle denotes fucose; MCC, microcrystalline cellulose; SPE, solid phase extraction; PGC, porous graphitized carbon

**Fig. 2:**
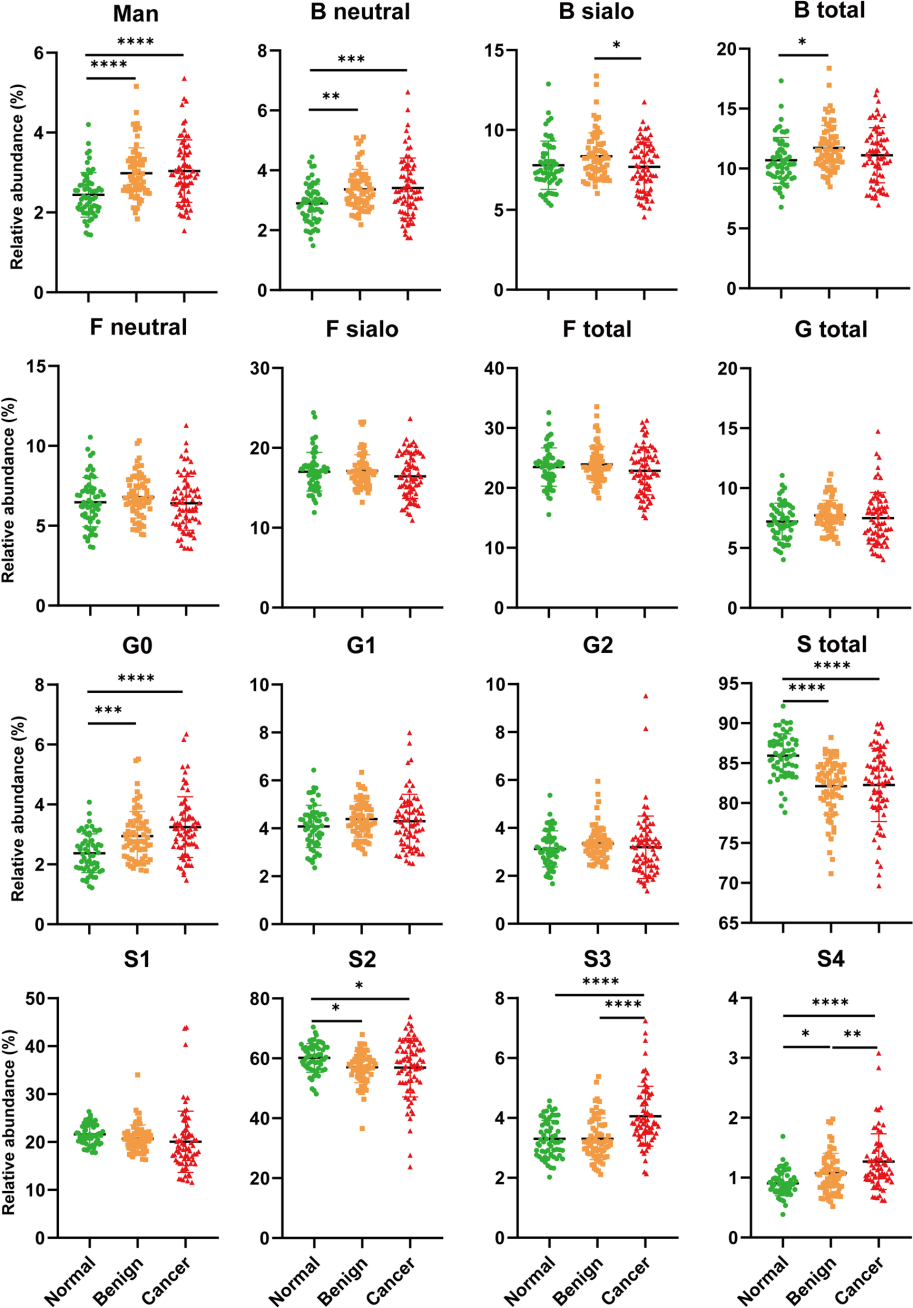
Serum *N*-glycans derived traits among normal, benign and cancer groups. Scatter plot was performed by one-way ANOVA with Bonferroni post hoc test. Significance is represented as not significant (n.s) *P* > 0.05, *P ≤ 0.05, **P ≤ 0.01, ***P ≤ 0.005, ****P ≤ 0.001

With the MALDI-MS based high-throughput glycomic technique, total serum *N*-glycome was profiled (Fig. 1, Table S3). Initially, quality control was performed using pooled serum samples, indicating the good repeatability and robustness of the technique (Fig S1). To explore the role of serum glycoforms in the development of OC, healthy controls and ovarian disease patients with benign neoplasms and OC patients were enrolled herein. Representative MALDI-MS spectra of serum *N*-glycome among normal and ovarian disease patients were displayed in Fig. S2. As illustrated in Fig. 2 and Table S4, statistical analysis showed that mannosylation was significantly increased in both benign and OC patients with *P* values less than 0.0001. Additionally, neutral bisection (B neutral) was also substantially elevated in benign and OC patients compared to normal group. Meanwhile, sialylated bisection (B sialo) in OC was lower than that in benign, and total bisection (B total) was increased in both benign and OC compared to normal, especially for benign group. This result may indicate the variety of bisection in OC progression, and the alteration in B neutral is noteworthy for OC monitoring. Moreover, agalactosylation (G0) was significantly increased from controls to OC patients with a gradual trend. On the contrary, total sialylation (S total) was remarkably reduced in ovarian disease patients with *P* value less than 0.0001. Interestingly, mono-sialylation (S1) and di-sialylation (S2) were decreased in ovarian patients, while tri-sialylation (S3) and tetra-sialylation (S4) were substantially changed in ovarian patients in the opposite trend. In addition, core-fucosylation (F) was not significantly changed in OC progression, which differs from many other cancers [30].

**Fig. 3:**
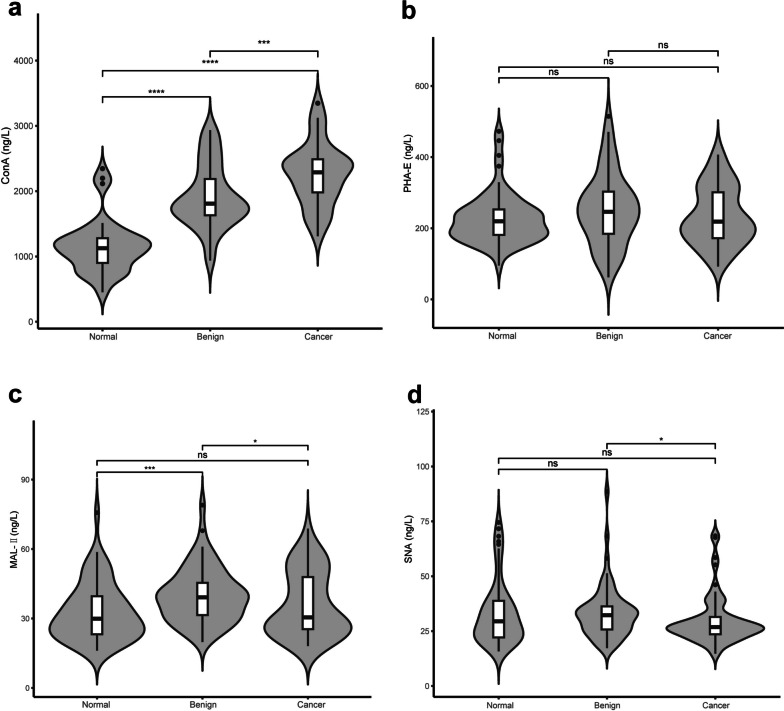
Lectin based ELISA assay analysis of high mannosylation (**a**), bisection (**b**), α2,3-linked sialylation (**c**) and α2,6-linked sialylation (**d**) in total serum from normal, benign and cancer groups. Scatter plots were generated by one-way ANOVA with Tukey’s post hoc test. Significance is represented as not significant (n.s) *P* > 0.05, *P ≤ 0.05, **P ≤ 0.01, ***P ≤ 0.005, ****P ≤ 0.001

Considering the strong association between OC progression and mannosylation, bisection and sialylation, ConA, SNA, MAL-II and PHA-E were employed in these orthogonal experiments (Fig. 3). It was observed that the antibody levels of ConA, PHA-E and MAL-II were increased in ovarian disease patients, and the level of SNA antibody was decreased in the patients’ serum, indicating the consistency between MALDI-MS data and lectin ELISA assay. Notably, linkage-specific sialylation in OC was differentially expressed, warranting further study on its role in cancer progression.

To understand the contribution of *N*-glycans to the variable glycan derived traits, individual serum *N*-glycans were analysed among controls and ovarian disease patients (Table S5, Fig. S3). It was found that all the *N*-glycans modified with oligomannose (H5N2, H6N2, H7N2, H8N2 and H9N2) were significantly increased in ovarian disease patients compared to healthy controls, especially for OC groups. Those oligomannosylated *N*-glycans gained at least moderately accurate AUC scores for discriminating ovarian disease patients from normal group (Table S5). In addition, all the hybrid *N*-glycans containing mannose residues (H5N3, H5N3F1, H6N3, H5N3S1 and H6N3S1) were significantly increased in OC patients, with at least moderately accurate AUC scores. These results suggest the vital role of oligomannosylation in OC progression. The agalactosylated glycans (H3N3, H3N3F1, H3N4F1, H3N5 and H3N5F1) were uniformly increased in ovarian disease patients, particularly for OC patients (Table S5, Fig. S2). Of note, most of the agalactosylated glycans showed a relatively good diagnostic performance for OC patients with AUC scores over 0.70. Three of the sialylated *N*-glycans (H4N4S1, H5N4S1F1 and H5N5S1F1) were significantly down-regulated in ovarian disease patients, reflecting differential mono-sialylation in OC progression. Notably, these three *N*-glycans were still significantly decreased from benign to cancer, implicating their inhibitory role in ovarian cancer progression. Similarly, two di-sialylated *N*-glycans (H5N4S2 and H5N5S2) were substantially decreased in OC patients, elucidating the alteration of S2 in OC patients. In contrast, the tri-sialylated *N*-glycans (H6N5S3F1, H7N6S3 and H7N6S3F1) and tetra-sialylated *N*-glycans (H7N6S4 and H7N6S4F1) were significantly increased in ovarian disease patients with a fair diagnostic performance. Additionally, the increase was still observed from benign to cancer patients, implicating the contribution of high sialylation to OC metastasis.

### Comparative study between benign and cancer patients

To explore the role of serum *N*-glycome in the process of precancerous lesions, further analysis was performed among benign neoplasms and OC patients with primary stages and advanced stages (Table 1, Fig. S4). We observed that agalactosylation was significantly increased in advanced stage of OC compared to benign neoplasms. Further analysis of individual *N*-glycans showed this alteration may be mainly reflected by glycan compositions of H3N3 and H3N4F1 with *P* values less than 0.05 (Table S6). Additionally, Tri-sialylation (S3) and tetra-sialylation (S4) of serum *N*-glycans were substantially increased in late stages of OC patients with *P* values less than 0.01. Notably, a significant increase of S3 was observed in the comparison between benign and early stage of OC patients, indicating the crucial role of S3 in contributing to precancerous lesions of OC. These alterations may be derived from the changes in the glycan compositions of H7N6S3, H7N6S3F1 and H7N6S4, with nearly moderately accurate AUC scores at 0.70 (Table S6, Fig. S5). However, high-mannosylation, bisection and core-fucosylation of serum glycoproteins were slightly changed in the metastatic development of OC. Interestingly, high-mannosylation was remarkably increased in both benign and OC patients compared to controls. Our data may reveal the potential of high-mannosylation for precancerous lesion prediction in OC.

**Table 1:** Serum *N*-glycome composition in benign neoplasm and ovarian cancer with different stages. Only the main glycan features describing glycome composition are shown. False discovery rate was controlled using Bonferroni (*Padj*). Man, mannosylation; G, galactose; F, fucose; B, bisecting GlcNAc; S, sialic acid. SD, standard deviation; CI, confidence interval

Dependent variable	Benign		Early Stages		Late Stages		Benign vs Early Stages		Benign vs Late Stages		Early Stages vs Late Stages	
	Mean (%)	SD (%)	Mean (%)	SD (%)	Mean (%)	SD (%)	95% CI	***Padj***	95% CI	***Padj***	95% CI	***Padj***
Man	2.98	0.64	2.95	0.77	3.08	0.79	(-0.37, 0.43)	1.00E+00	(-0.43, 0.23)	1.00E+00	(-0.56, 0.29)	1.00E+00
G0	2.94	0.82	2.96	1.01	3.41	0.99	(-0.52, 0.49)	1.00E+00	(-0.89, -0.05)	**2.40E-02**	(-0.99, 0.09)	1.38E-01
G1	4.38	0.74	4.07	1.02	4.42	1.18	(-0.22, 0.84)	4.68E-01	(-0.47, 0.40)	1.00E+00	(-0.91, 0.22)	4.18E-01
G2	3.35	0.69	3.08	0.90	3.25	1.48	(-0.32, 0.85)	8.25E-01	(-0.39, 0.57)	1.00E+00	(-0.79, 0.45)	1.00E+00
G total	7.73	1.25	7.16	1.83	7.67	2.32	(-0.41, 1.56)	4.72E-01	(-0.75, 0.87)	1.00E+00	(-1.57, 0.53)	6.98E-01
F neutral	1.32	0.16	6.27	1.70	6.48	1.68	(-0.32, 1.37)	3.95E-01	(-0.38, 1.02)	8.05E-01	(-1.11, 0.69)	1.00E+00
F sialo	17.10	2.03	15.79	3.01	16.79	2.60	(-0.04, 2.66)	6.00E-02	(-0.80, 1.43)	1.00E+00	(-2.44, 0.44)	2.86E-01
F total	23.90	3.00	22.06	4.44	23.27	3.68	(-0.12, 3.80)	7.40E-02	(-0.99, 2.25)	1.00E+00	(-3.30, 0.89)	4.96E-01
B neutral	3.36	0.66	3.28	1.01	3.48	1.00	(-0.39, 0.56)	1.00E+00	(-0.51, 0.27)	1.00E+00	(-0.71, 0.31)	1.00E+00
B sialo	8.35	1.46	7.52	1.59	7.79	1.54	(-0.01, 1.68)	5.20E-02	(-0.13, 1.26)	1.55E-01	(-1.17, 0.63)	1.00E+00
B total	11.72	1.88	10.80	2.45	11.27	2.22	(-0.25, 2.09)	1.79E-01	(-0.52, 1.41)	8.03E-01	(-1.73, 0.78)	1.00E+00
S1	20.71	2.87	20.50	4.82	19.78	7.16	(-2.57, 2.98)	1.00E+00	(-1.37, 3.22)	9.92E-01	(-2.25, 3.68)	1.00E+00
S2	57.02	5.21	57.48	7.69	56.56	10.83	(-4.85, 3.92)	1.00E+00	(-3.16, 4.09)	1.00E+00	(-3.76, 5.61)	1.00E+00
S3	3.31	0.69	3.86	0.99	4.17	1.00	(-1.03, -0.07)	**1.80E-02**	(-1.26, -0.47)	**1.17E-06**	(-0.83, 0.19)	3.96E-01
S4	1.07	0.33	1.15	0.48	1.33	0.45	(-0.30, 0.15)	1.00E+00	(-0.44, -0.07)	**3.00E-03**	(-0.42, 0.06)	2.09E-01
S total	82.11	3.51	82.99	4.35	81.84	4.69	(-3.15, 1.39)	1.00E+00	(-1.61, 2.14)	1.00E+00	(-1.28, 3.57)	7.61E-01

Furthermore, we observed that the alterations in levels of CA 125 were strongly correlated with glycosylation changes in serum glycoproteins (Table S7, Fig. S6). Specifically, sialylation was negatively correlated with CA 125 (*r* = -0.26, *P* = 0.023). Agalactosylation (*r* = 0.23, *P* = 0.05) and mono-galactosylation (*r* = 0.28, *P* = 0.02) were positively correlated with CA 125. In addition, high mannosylation (*r* = 0.29, *P* = 0.01) of serum glycoproteins were positively correlated with CA 125. However, HE4 was not correlated with any N-glycosylation of serum glycoproteins, demonstrating the independent alteration of this routine criteria.

### Evaluation of diagnostic performance of serum *N*-glycome for ovarian cancer

**Fig. 4:**
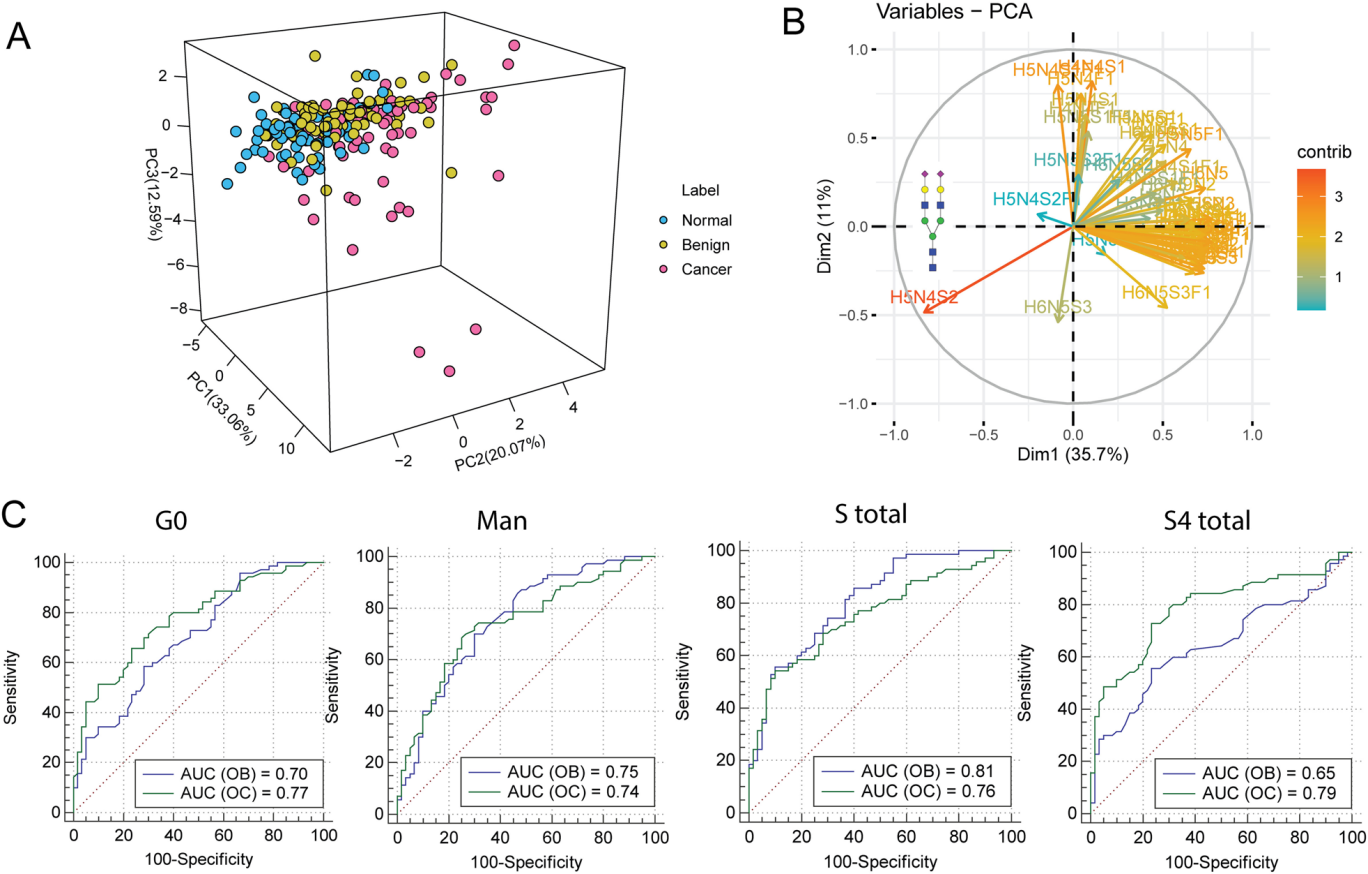
Classification of ovarian disease patients using serum *N*-glycans. **A** Three-dimensional principal component analysis (3D-PCA) of serum *N*-glycome for distinguishing ovarian disease patients from controls; (**B**) Plot of variable importance for the projection (VIP) showing the importance of the glycan variables; (**C**) ROC analysis of significantly different glycan features for the prediction of OC

 Due to the close link between the significantly changed serum protein N-glycosylation and ovarian cancer, discriminative models were employed to evaluate the diagnostic performance. Using the significantly different glycans as the variables in the aforementioned comparison, unsupervised principal component analysis (PCA) was performed to assess the differentiation between controls and ovarian disease patients. It was apparent that normal group was almost completely distinguished from both benign and OC patients with the accuracy of 65.72% (Fig. 4A), indicating the good performance of serum *N*-glycans for screening OC patients from healthy controls. Plot of variable importance for the projection showed that several of the variables contributed to the differentiation between controls and ovarian disease patients, such as the disialylated biantennary *N*-glycan (H5N4S2) (Fig. 4B).

**Fig. 5:**
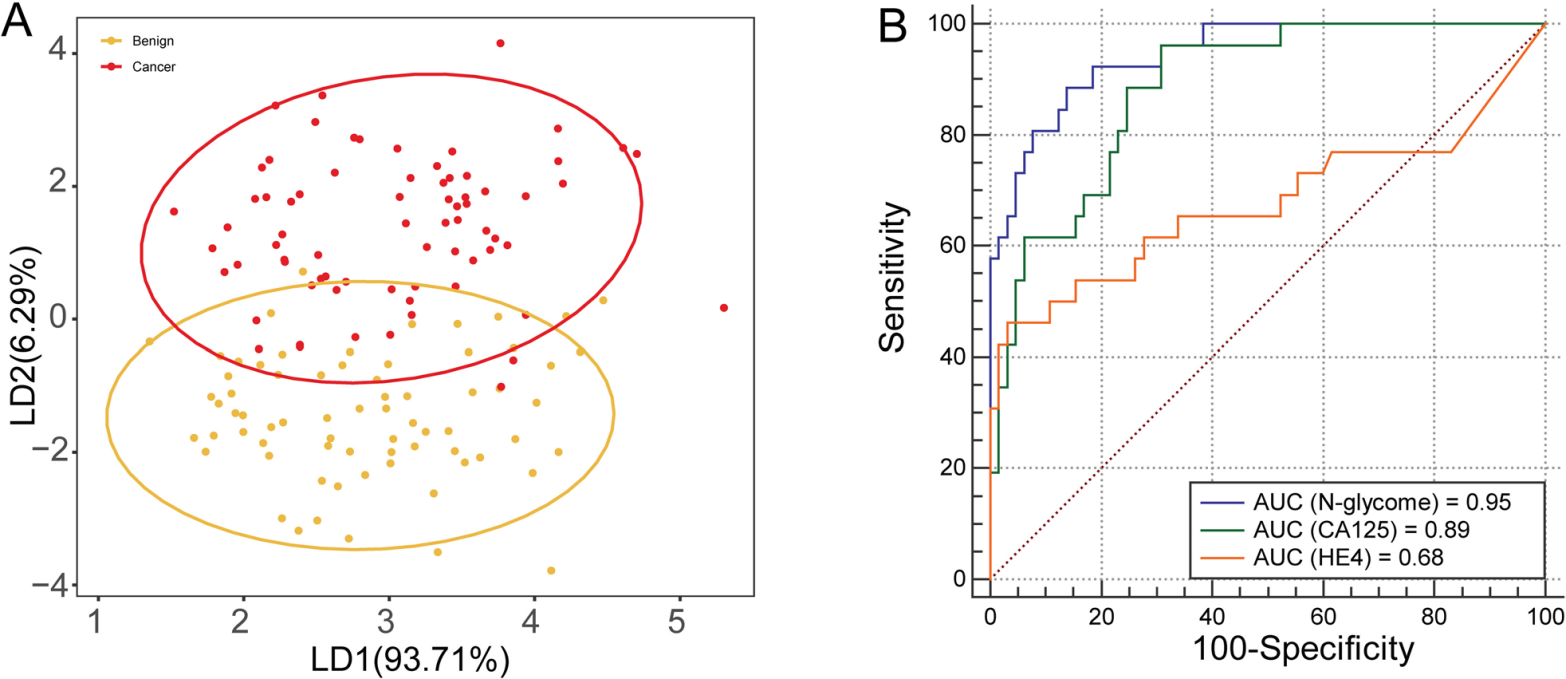
Discriminative models illustrating the power of serum *N*-glycome for precancerous lesion prediction. **A** Linear discriminant analysis (LDA) of serum *N*-glycans for distinguishing cancer patients from benign neoplasms; **B** ROC analysis of serum *N*-glycome, HE4 and CA125 between benign patients and OC patients with early stage

 To elucidate the diagnostic performance of the glycan derived traits, ROC curve was performed in the comparison between normal and ovarian disease patients. Our data showed that glycan features of high-mannosylation, agalactosylation and sialylation gained at least moderately accurate AUC scores for stratifying benign group from healthy controls (Fig. 4C). In addition, those three types of glycan features could distinguish OC patients from controls with at least accurate AUC scores, suggesting their potential for early diagnosis of OC. Furthermore, we explored the ability of significantly changed *N*-glycans in serum to discriminate OC patients from benign neoplasms. Interestingly, apparent discrimination was observed using serum *N*-glycome as analyzed by unsupervised LDA (Fig. 5A). In addition, those significant *N*-glycans (*N*-glycome) gained a highly accurate AUC score for discriminating OC patients from benign, demonstrating the efficacy of serum *N*-glycome for precancerous lesions prediction (Fig. 5B). Moreover, it is noteworthy that the glycan panels showed a higher diagnostic performance than HE4, and almost equal to CA 125, providing a new diagnostic tool for OC.

### Serum *N*-glycome based classification model for OC

**Fig. 6:**
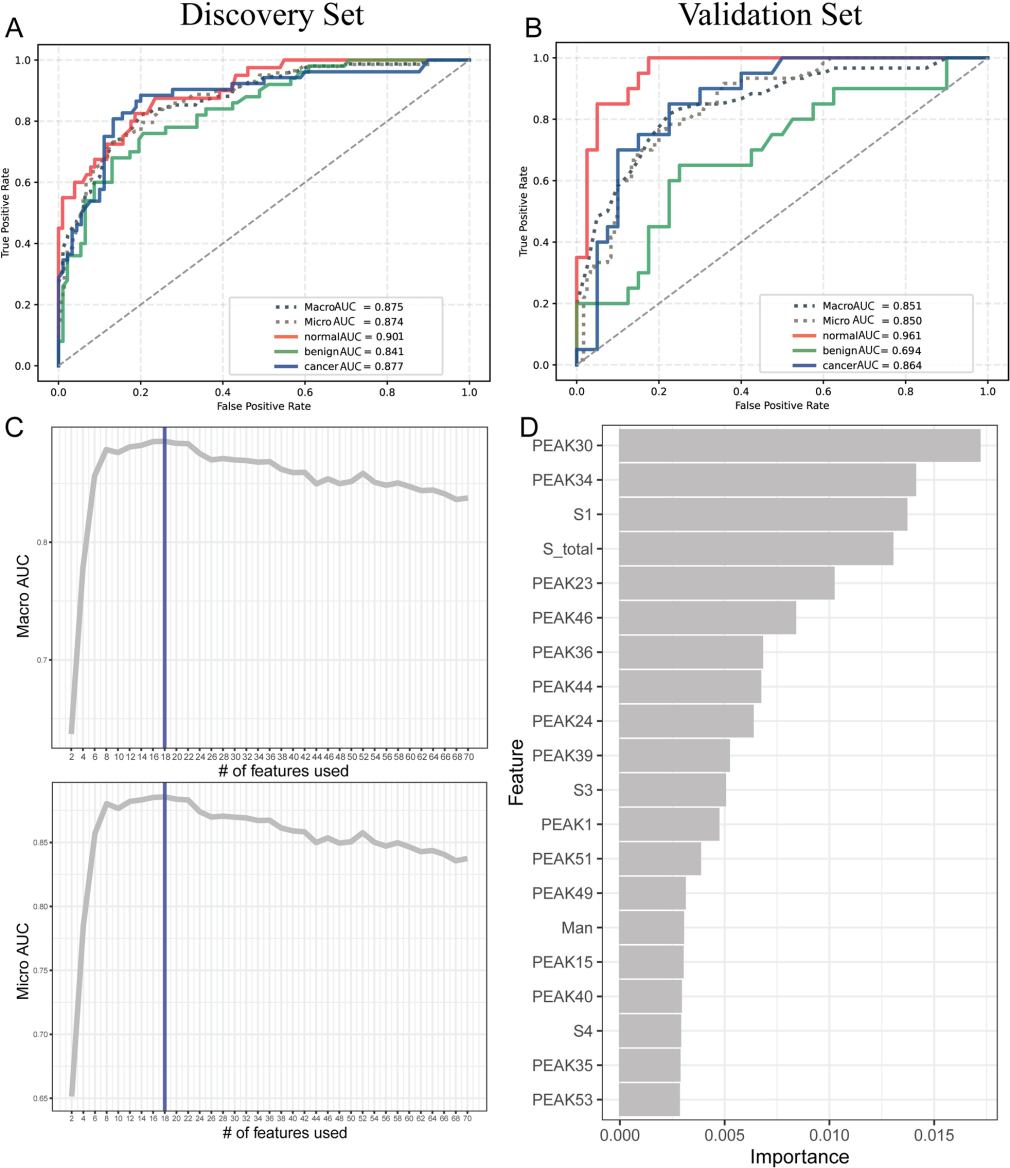
Bioinformatics analysis of the diagnostic performance of serum *N*-glycome in OC. **A**-**B** Three-classification ROC analysis of serum *N*-glycome among normal, benign and cancer groups by random forest in both discovery and validation sets; **C**-**D** The importance of features for the contribution to the classification

To further elucidate the diagnostic performance of serum *N*-glycome for OC diagnosis, a machine learning based model was built by reanalyzing the raw data. Three-classification model showed accurate AUC scores for all ROC curves in discovery set, demonstrating the good diagnostic performance for ovarian disease patients using serum *N*-glycome (Fig. 6A). Notably, the results were elucidated by ROC analysis in validation set except for the classification between benign and the other groups (Fig. 6B). Nevertheless, ROC curve analysis of benign versus normal and cancer groups yielded an AUC score of 0.69, nearly to moderate accuracy. The optimal discriminative performance for the classification between controls and ovarian disease patients was obtained using top 18 features (Fig. 6C-D). Significantly, most of those top 18 features were matched with the candidate serum *N*-glycan for OC diagnosis mentioned above, indicating the reliability of the findings in this study.

## Discussion

Ovarian cancer represents the most lethal gynecological carcinoma that is frequently diagnosed at an advanced TNM stage. Benign neoplasms of the ovary are characterized by serous cyst or ovarian adenoma, which could accelerate the stepwise progression toward ovarian malignancy. Although several pathological features in precursor lesion are shared with OC tumors, the molecular events involved in early ovarian oncogenesis are still largely unknown. Additionally, some controversies concerning precursor lesions encountered in the study of early ovarian cancer, impeding the diagnosis of precancerous lesions [31]. Blood or serum test is one of the most frequent and convenient approach to disease screening in clinic. The human epididymis protein 4 (HE4) and carbohydrate antigen 125 (CA 125) are the most widely used serum biomarkers in OC screening, while both of them suffer from relatively low specificity or sensitivity for OC diagnosis [32]. Therefore, it is necessary to discover complementary molecular markers for OC screening and early diagnosis. In the new emerging paradigm of clinical studies, glycomedicine plays an indispensable role in cancer diagnosis, therapy development and disease monitoring [33]. Many studies have demonstrated the crucial role of serum protein *N*-glycosylation in cancer progression [34], while a few studies have focused on ovarian cancer. Based on the high-throughput analytical assay as described previously [20], serum *N*-glycome profile was characterized in this study, including healthy controls, benign neoplasm and OC patients at different TNM stages.

Our data showed significantly higher mannosylation of serum glycoproteins could distinguish both benign and OC patients from controls with moderately accurate diagnostic merits. Interestingly, altered mannosylation of serum was positively correlated with CA 125, the routine biomarker for OC diagnosis, indicating its positive impact on inflammatory processes in OC development. To our knowledge, this was the first observation with relatively large sample scale since previous study showed the increased mannosylated glycan (M9) of serum in OC patient [35]. However, down-regulated high-mannose *N*-glycans in OC patients have been reported in other studies [36], even though the slightly increase was found between early stage and controls [37]. This inconsistency may result from the sample size, or the heterogeneity of study populations [38], or interlaboratory glycosylation analysis methods [39], warranting further investigations with larger scale cohorts. Recently, the elevated high-mannose *N*-glycans were also exhibited in mouse ovarian cancer tissue [40], demonstrating the crucial role of mannosylation in OC development. Moreover, significantly increased high-mannose glycans were reported in many other cancers, such as cholangiocarcinoma [41], breast cancer [42, 43] and periampullary adenocarcinoma [44], suggesting that the altered mannosylation of serum is not specific to OC.

Agalactosylation was found to be significantly increased in OC patients and benign compared to controls with both moderately accurate diagnostic merits, in addition to the increase from benign neoplasm to late stages. These alterations were reflected by the significantly changed agalactosylated *N*-glycans, which was in agreement with a previous study [45]. Additionally, many literatures have reported the strong association between significantly increased agalactosylation of IgG and OC patients [46–48], chiefly implicating the main contribution of IgG to the altered glycosylation of serum glycoprotein. The observations are most likely due to the pro-inflammatory process in tumor progression and metastasis [49]. The strong positive correlation between agalactosylation of serum and CA 125 may further elucidate the critical role of aglactosylation in the promotion of OC progression. In addition, adding galactosylation to the diagnostic index would improve the accuracy of OC diagnosis.

Similarly, bisecting GlcNAcylated *N*-glycans, particularly for neutral bisection, were up-regulated in both of benign and OC patients, which was agreement with previous study [17]. Further bioinformatics analysis by GEPIA2 showed the increased expression of MGAT3 that encodes *N*-acetylglucosaminyltransferase III (GnT-III) [50], verifying the altered bisection in OC progression. Notably, it was found that up-regulated GnT-III activates Notch signaling and drives stem cell expansion to promote the malignant development of OC [51], demonstrating the therapeutic potential of inhibiting GnT-III for OC intervention or treatment. In contrast, sialylated glycans of bisecting GlcNAc (B sialo) was significantly decreased in OC patients compared to benign neoplasms, suggesting the inhibitory role of B sialo in OC progression. This result agreed to some extent with previous studies showing that up-regulated expression of GnT-III inhibits tumor metastasis [52, 53]. Additionally, this effect may be attributed to the sialylation due to the strong association between decreased sialylation and OC progression. Overall, the effector function of B sialo results from the compromise effect of both sialylation and bisection, thus it is ambiguous to dissect the role of sialylated bisection in OC.

Sialylation of serum glycoproteins in OC progression was significantly changed herein, mainly reflected by triantennary and tetraantennary sialylated *N*-glycans in late stage of OC patients. Besides, the increased expression of *ST6GAL1* elucidated the alteration of sialylation in OC [50]. The result agreed with previous literature about serum protein N-glycosylation analysis in OC [36]. Interestingly, high-grade tumors in several other types of cancers, such as lung cancer, neuoblastoma and gliomas, were associated with increased sialylation [54], indicating the potential specificity of serum sialylation to OC. It is worth noting that an increase in tri- and tetra-antennary structures could induce cancer progression due to its positive correlation with branching GlcNAc *N*-glycans [55], implicating the considerable effect of high-branching sialylation on OC progression. Therefore, multi-antennary sialylated *N*-glycans are likely promising indictors for OC diagnosis and precancerous lesions.

In conclusion, serum *N*-glycome profiles in ovarian cancer onset and progression were comprehensively characterized by a high-throughput technique. Importantly, a new classification model using serum *N*-glycans was employed for differentiation between healthy, benign neoplasms and ovarian cancer. To our knowledge, this is the first study demonstrating that serum *N*-glycome can be exploited as biomarker tool for the prediction of precancerous lesions of OC, providing significant insight into the regulation of *N*-glycosylation in OC progression. Notably, the addition of noninvasive serum *N*-glycan signatures to the current risk assessment tools may improve the accuracy of early diagnosis for OC, warranting further investigations with larger scale cohorts. Considering the complexity of human serum glycoproteins, further protein- and site-specific glycosylation profiling in OC is vital for our understanding of the molecular mechanism regulated by glycosylation mediated signaling.

### Supplementary Information

**Additional file 1: Table S1.** Demographic description of study populations in this study. **Table S2.** Demographic description of study populations split into two independent sets in bioinformatics analysis. **Table S3.** MALDI-MS spectra of *N*-glycans identified in human serum. **Table S4.** Description of the significantly changed glycan derived traits in OC.** Table S5.** Statistical analysis of individual glycans among controls, benign neoplasms and OC patients. **Table S6.** Statistical analysis of individual glycans between benign neoplasm and different stages of OC patients. **Table S7.** Pearson correlation between glycan derived traits and routine clinical criteria for OC. **Figure S1.** Evaluation of the reproducibility for the quantitation of serum *N*-glycome by MALDI-MS.** Figure S2.** Representative MALDI-MS spectra of serum *N*-glycans in the comparison between controls, benign neoplasm and different stages of OC. **Figure S3.** Violin plots of the individual *N*-glycans in the comparison between normal and ovarian disease patients. **Figure S4.** Differentially expressed serum *N*-glycan derived traits in the comparison between benign and different stages of OC patients. **Figure S5.** Violin plots of the individual *N*-glycans in the comparison between benign and different stages of OC patients. **Figure S6.** Pearson correlation between serum glycosylation and CA 125.

## Data Availability

The mass spectrometry glycomic data have been deposited at the GlycoPOST archive with the data set identifier GPST000364.
