# Supplementary material for: Noninvasive serum N-glycans associated with ovarian cancer diagnosis and precancerous lesion prediction
Source: J Ovarian Res. 2024 Jan 27;17:26. doi: 10.1186/s13048-024-01350-2 (PMC10821556; doi:10.1186/s13048-024-01350-2)
Supplement: Supplementary file 1 — Additional file 1: Table S1. Demographic description of study populations in this study. Table S2. Demographic description of study populations split into two independent sets in bioinformatics analysis. Table S3. MALDI-MS spectra of N-glycans identified in human serum. Table S4. Description of the significantly changed glycan derived traits in OC. Table S5. Statistical analysis of individual glycans among controls, benign neoplasms and OC patients. Table S6. Statistical analysis of individual glycans between benign neoplasm and different stages of OC patients. Table S7. Pearson correlation between glycan derived traits and routine clinical criteria for OC. Figure S1. Evaluation of the reproducibility for the quantitation of serum N-glycome by MALDI-MS. Figure S2. Representative MALDI-MS spectra of serum N-glycans in the comparison between controls, benign neoplasm and different stages of OC. Figure S3. Violin plots of the individual N-glycans in the comparison between normal and ovarian disease patients. Figure S4. Differentially expressed serum N-glycan derived traits in the comparison between benign and different stages of OC patients. Figure S5. Violin plots of the individual N-glycans in the comparison between benign and different stages of OC patients. Figure S6. Pearson correlation between serum glycosylation and CA 125. [file 13048_2024_1350_MOESM1_ESM.docx]

**Noninvasive serum *N*-glycans associated with ovarian cancer diagnosis and precancerous lesion prediction**

Si Liu,^1, 2, ‡^ Chang Tu,^1, ‡^ Haobo Zhang,^1^ Hanhui Huang,^1^ Yuanyuan Liu, ^1^ Yi Wang,^3^

Liming Cheng,^3^ Bi-Feng Liu, ^1^ Kang Ning,^1, *^ Xin Liu ^1, **^

^1^The Key Laboratory for Biomedical Photonics of MOE at Wuhan National Laboratory for Optoelectronics-Hubei Bioinformatics & Molecular Imaging Key Laboratory, Systems Biology Theme, Department of Biomedical Engineering, College of Life Science and Technology, Huazhong University of Science and Technology, Wuhan 430074, China

^2^Department of Epidemiology and Health Statistics, School of Public Health, Fujian Medical University, Fuzhou, China.

^3^Department of Laboratory Medicine, Tongji Hospital, Tongji Medical College, Huazhong University of Science and Technology, Wuhan, China

‡These authors contributed equally.

*Corresponding author

Kang Ning: [ningkang@hust.edu.cn](mailto:ningkang@hust.edu.cn)

Xin Liu: [xliu@mail.hust.edu.cn](mailto:xliu@mail.hust.edu.cn)

**Contents**

**Table S1** Demographic description of study populations in this study.

**Table S2** Demographic description of study populations split into two independent sets in bioinformatics analysis.

Table S3 MALDI-MS spectra of *N*-glycans identified in human serum.

**Table S4** Description of the significantly changed glycan derived traits in OC.

**Table S5** Statistical analysis of individual glycans among controls, benign neoplasms and OC patients.

**Table S6** Statistical analysis of individual glycans between benign neoplasms and different stages of OC patients.

**Table S7** Pearson correlation between glycan derived traits and routine clinical criteria for OC.

**Figure S1**. Evaluation of the reproducibility for the quantitation of serum N-glycome by MALDI-MS.

**Figure S2** Representative MALDI-MS spectra of serum *N*-glycans in the comparison between controls, benign neoplasm and different stages of OC.

**Figure S3** Violin plots of the individual *N*-glycans in the comparison between normal and ovarian disease patients.

**Figure S5** Differentially expressed serum *N*-glycan derived traits in the comparison between benign and different stages of OC patients.

**Figure S6** Violin plots of the individual *N*-glycans in the comparison between benign and different stages of OC patients.

**Figure S7** Pearson correlation between serum glycosylation and CA 125.

**Table S1** Demographic description of study populations in this study. IQR, inter-quartile range; TNM, tumor node metastasis. *P* values of age resulted from the one-factor ANOVA analysis; *P* value of CA125 or HE4 resulted from Mann-Whitney U test. P value less than 0.05 was set as significant.

**Annotation:** a, comparison between heathy controls and benign ovarian disease patients;

b, comparison between heathy controls and ovarian cancer cohorts;

c, comparison between benign ovarian disease patients and ovarian cancer cohorts.

**Table S2** Demographic description of study populations split into two independent sets in bioinformatics analysis. IQR, inter-quartile range; TNM, tumor node metastasis.

**Table S3** MALDI-MS spectra of *N*-glycans identified in human serum. Symbols as Figure S1.

| No. Peak | Glycan (*m/z*) [M+Na] | | | Chemical composition | Glycan structure |
| --- | --- | --- | --- | --- | --- |
|  | Theoretical | Observed | Mass error (ppm) |  |  |
| 1 | 1136.3964 | 1136.5122 | 101.9011 | H3N3 | 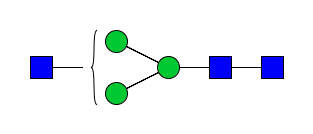 |
| 2 | 1257.4226 | 1257.5424 | 95.3379 | H5N2 | 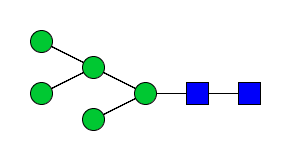 |
| 3 | 1282.4543 | 1282.5777 | 96.2678 | H3N3F1 | 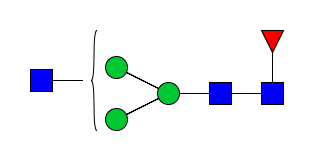 |
| 4 | 1298.4492 | 1298.5689 | 92.2408 | H4N3 | 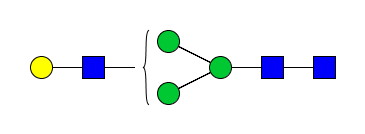 |
| 5 | 1339.4757 | 1339.5906 | 85.8530 | H3N4 | 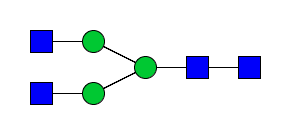 |
| 6 | 1419.4755 | 1419.6035 | 90.1854 | H6N2 | 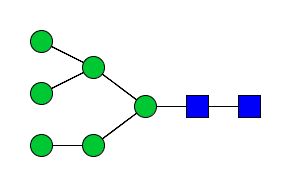 |
| 7 | 1444.5071 | 1444.6508 | 99.5350 | H4N3F1 | 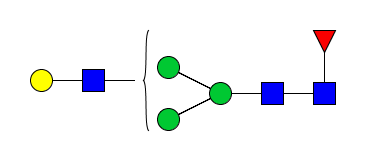 |
| 8 | 1460.5020 | 1460.6402 | 94.6654 | H5N3 | 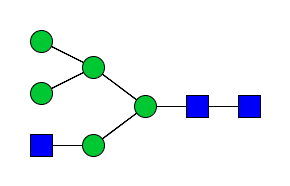 |
| 9 | 1485.5337 | 1485.6589 | 84.3037 | H3N4F1 | 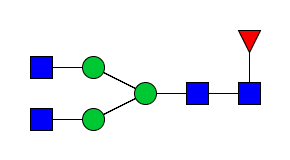 |
| 10 | 1501.5286 | 1501.9689 | 293.2971 | H4N4 | 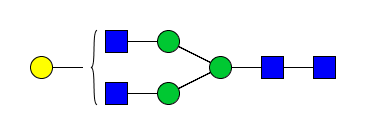 |
| 11 | 1542.5551 | 1542.6962 | 91.5293 | H3N5 | 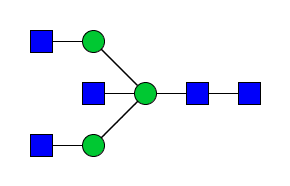 |
| 12 | 1581.5283 | 1581.6546 | 79.8993 | H7N2 | 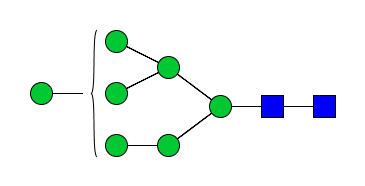 |
| 13 | 1602.5446 | 1602.7058 | 100.5969 | H4N3S1 | 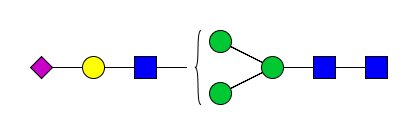 |
| 14 | 1606.5599 | 1606.7276 | 104.4225 | H5N3F1 | 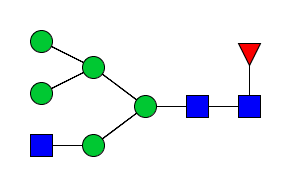 |
| 15 | 1622.5548 | 1622.7012 | 90.2860 | H6N3 | 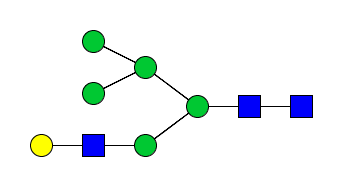 |
| 16 | 1647.5865 | 1647.7176 | 79.6019 | H4N4F1 | 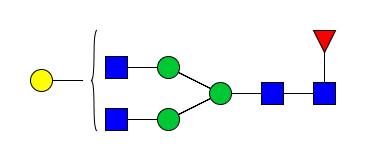 |
| 17 | 1663.5814 | 1663.7122 | 78.6736 | H5N4 | 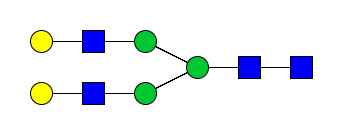 |
| 18 | 1688.6130 | 1688.7461 | 78.8185 | H3N5F1 | 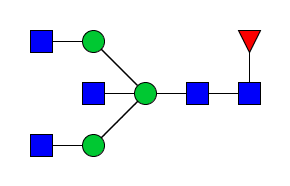 |
| 19 | 1704.6079 | 1704.7513 | 84.1501 | H4N5 | 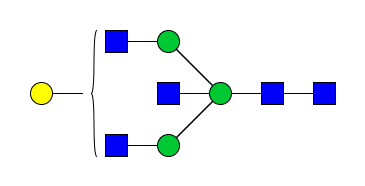 |
| 20 | 1743.5811 | 1743.7379 | 89.9385 | H8N2 | 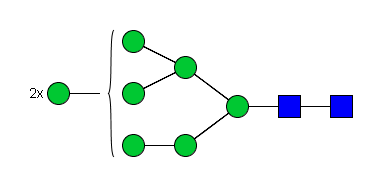 |
| 21 | 1748.6025 | 1748.7750 | 98.6639 | H4N3S1F1 | 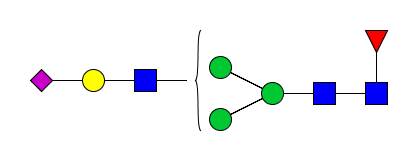 |
| 22 | 1764.5974 | 1764.7628 | 93.7421 | H5N3S1 | 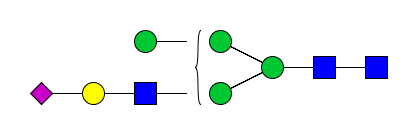 |
| 23 | 1805.6240 | 1805.7808 | 86.8863 | H4N4S1 | 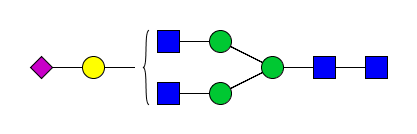 |
| 24 | 1809.6393 | 1809.7739 | 74.3938 | H5N4F1 | 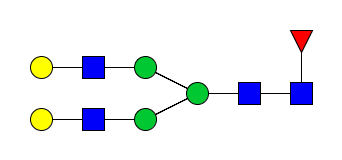 |
| 25 | 1850.6659 | 1850.8009 | 72.9483 | H4N5F1 | 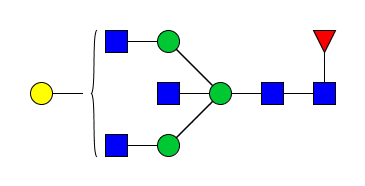 |
| 26 | 1866.6608 | 1866.7924 | 70.5431 | H5N5 | 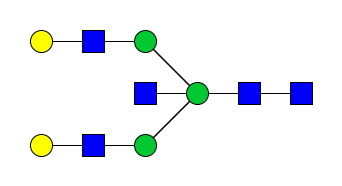 |
| 27 | 1905.6339 | 1905.7696 | 71.2377 | H9N2 | 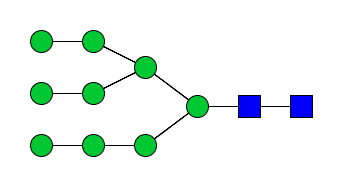 |
| 28 | 1926.6503 | 1926.8273 | 91.9176 | H6N3S1 | 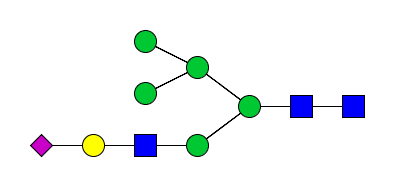 |
| 29 | 1951.6819 | 1951.8498 | 86.0560 | H4N4S1F1 | 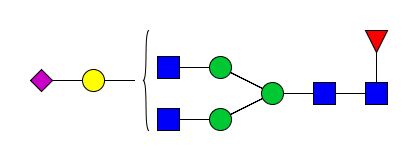 |
| 30 | 1967.6768 | 1967.8369 | 81.3721 | H5N4S1 | 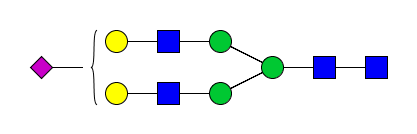 |
| 31 | 2008.7034 | 2008.8608 | 78.3789 | H4N5S1 | 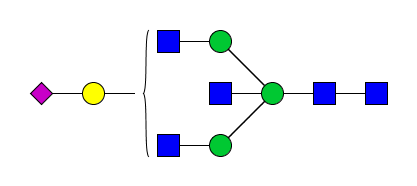 |
| 32 | 2012.7187 | 2012.8475 | 64.0099 | H5N5F1 | 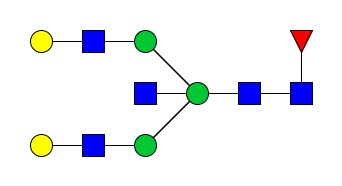 |
| 33 | 2028.7136 | 2028.8455 | 65.0565 | H6N5 | 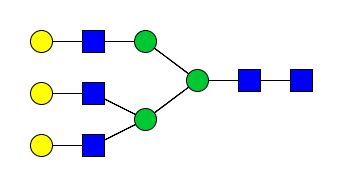 |
| 34 | 2113.7347 | 2113.9981 | 77.3479 | H5N4S1F1 | 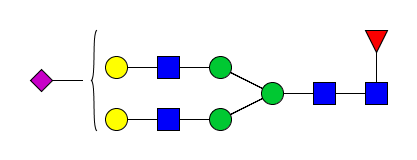 |
| 35 | 2154.7613 | 2154.9291 | 77.9200 | H4N5S1F1 | 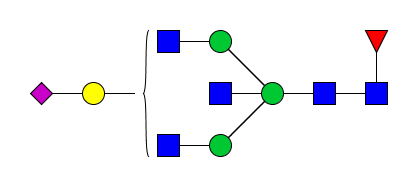 |
| 36 | 2170.7562 | 2170.9150 | 73.1722 | H5N5S1 | 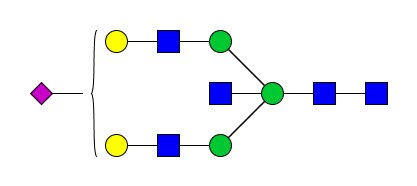 |
| 37 | 2271.7722 | 2271.9582 | 81.8973 | H5N4S2 | 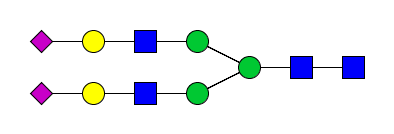 |
| 38 | 2316.8141 | 2316.9721 | 68.2265 | H5N5S1F1 | 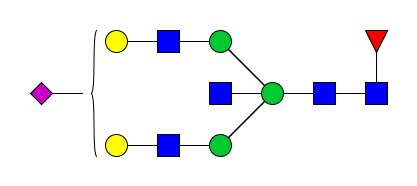 |
| 39 | 2332.8090 | 2332.9648 | 66.8053 | H6N5S1 | 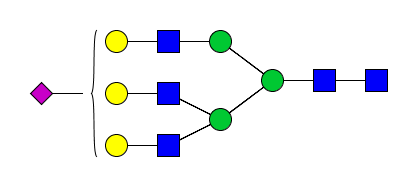 |
| 40 | 2417.8301 | 2418.0180 | 77.7416 | H5N4S2F1 | 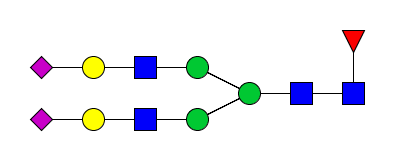 |
| 41 | 2474.8516 | 2475.0683 | 87.5847 | H5N5S2 | 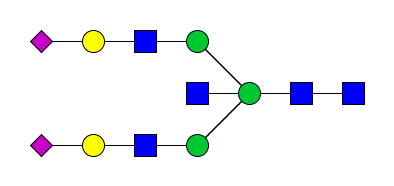 |
| 42 | 2478.8669 | 2479.0122 | 58.6183 | H6N5S1F1 | 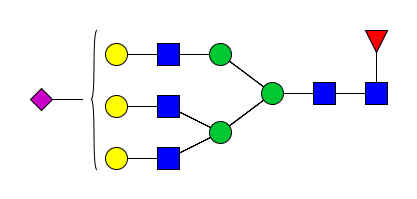 |
| 43 | 2620.9095 | 2621.0949 | 70.7659 | H5N5S2F1 | 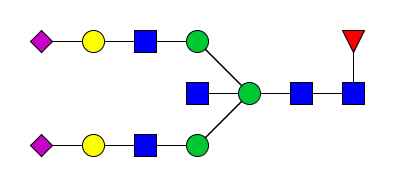 |
| 44 | 2636.9044 | 2637.0761 | 65.1415 | H6N5S2 | 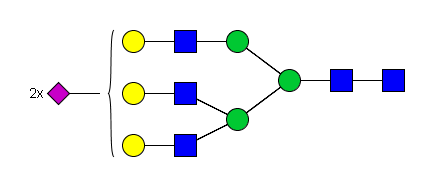 |
| 45 | 2697.9412 | 2698.0500 | 40.3452 | H7N6S1 | 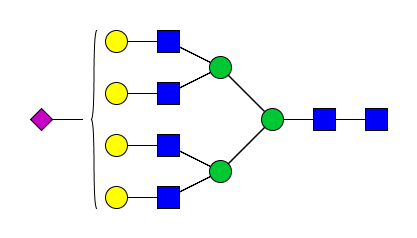 |
| 46 | 2782.9623 | 2783.1296 | 60.1298 | H6N5S2F1 | 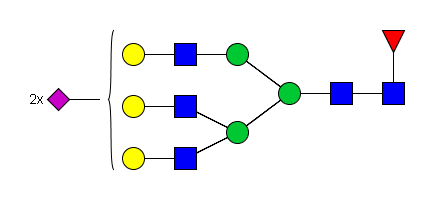 |
| 47 | 2940.9998 | 2941.2933 | 65.8140 | H6N5S3 | 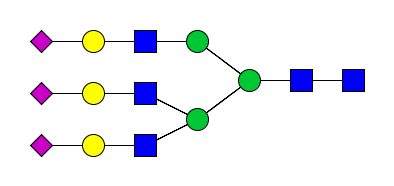 |
| 48 | 3002.0366 | 3002.2250 | 62.7900 | H7N6S2 | 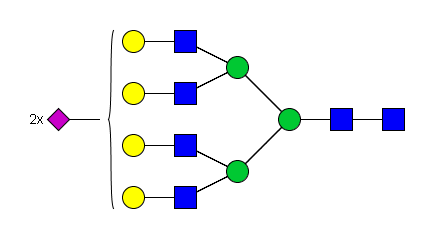 |
| 49 | 3087.0577 | 3087.2402 | 59.1288 | H6N5S3F1 | 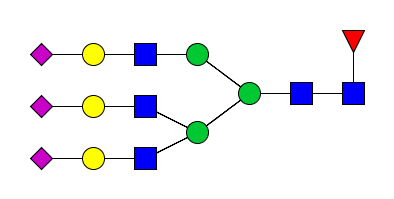 |
| 50 | 3148.0948 | 3148.3962 | 95.7405 | H7N6S2F1 | 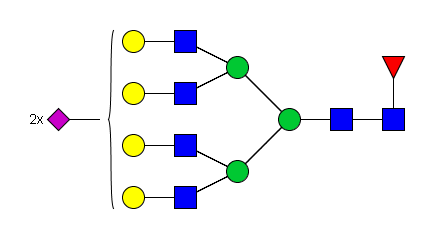 |
| 51 | 3306.1320 | 3306.2927 | 48.6142 | H7N6S3 | 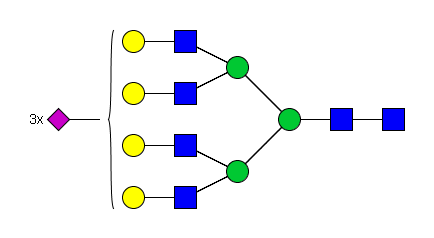 |
| 52 | 3452.1899 | 3452.3483 | 45.9097 | H7N6S3F1 | 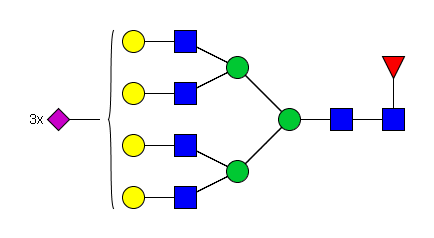 |
| 53 | 3610.2275 | 3610.3598 | 36.6633 | H7N6S4 | 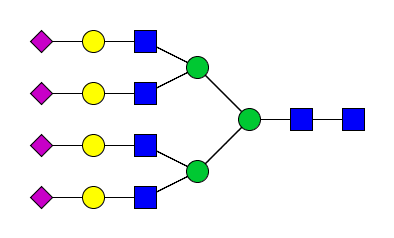 |
| 54 | 3756.2854 | 3756.7731 | 129.8357 | H7N6S4F1 | 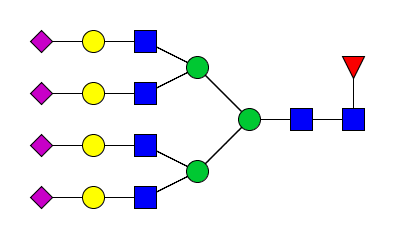 |

**Table S4** Description of the significantly changed glycan derived traits in OC. SD, standard deviation; CI, confident interval; *Padj*, adjusted *P* value.

**Table S5** Statistical analysis of individual glycans among controls, benign neoplasm and OC patients. Symbols as Figure S1.

**Table S6** Statistical analysis of individual glycans between benign neoplasm and different stages of OC patients. Symbols as Figure S1.

**Table S7** Pearson correlation between glycan derived traits and routine clinical criteria for OC. Significant difference was underscored with bold.

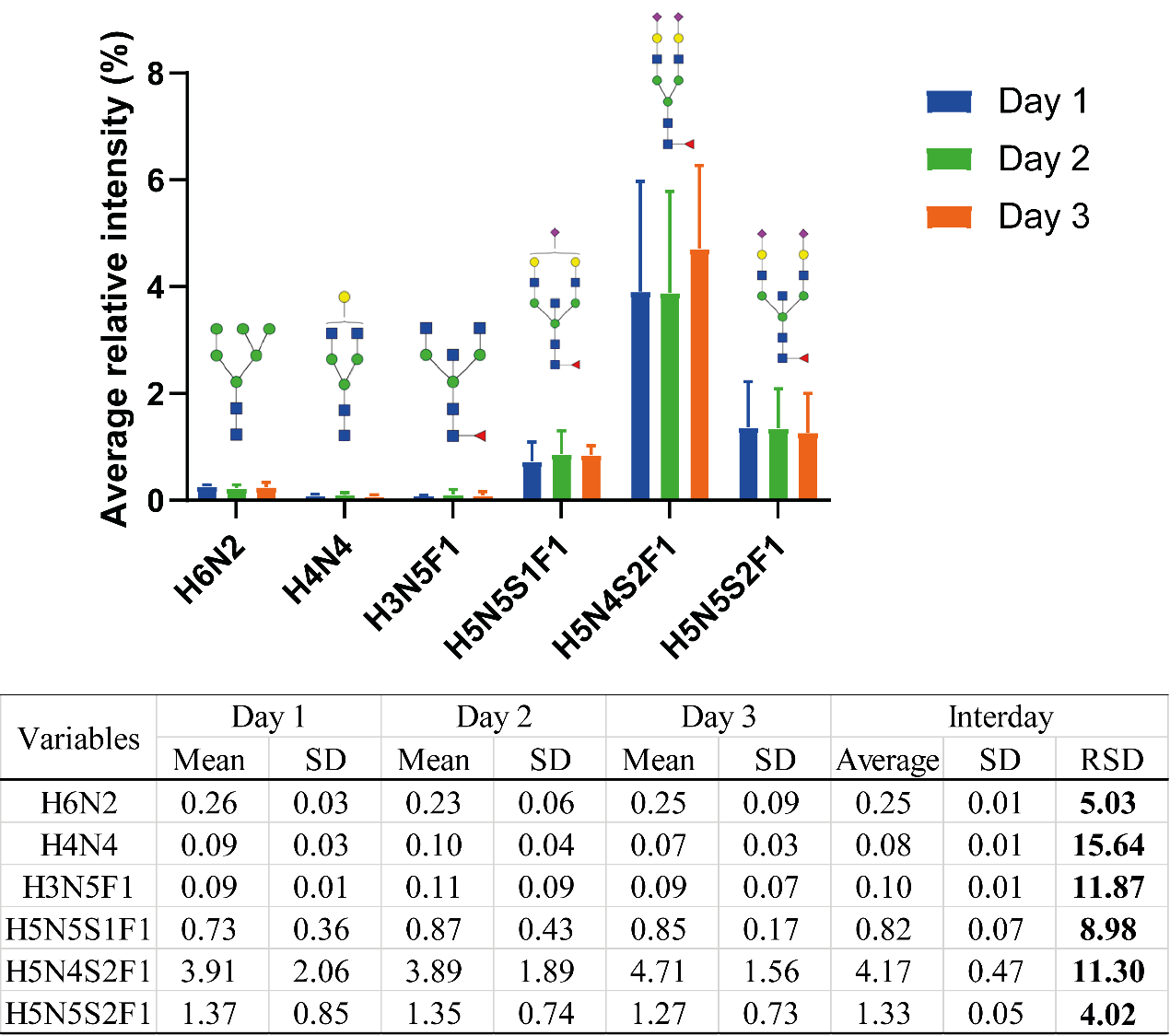


**Fig S1** Evaluation of the reproducibility for the quantitation of serum *N*-glycome by MALDI-MS. H, hexose; N, N-acetylglucosamine; F, fucose; S, sialic acid; SD, standard deviation; RSD, relative standard deviation. Symbols were presented as follows: blue square, N-acetylglucosamine; green circle, mannose; yellow circle, galactose; purple diamond, sialic acid; red triangle, fucose.


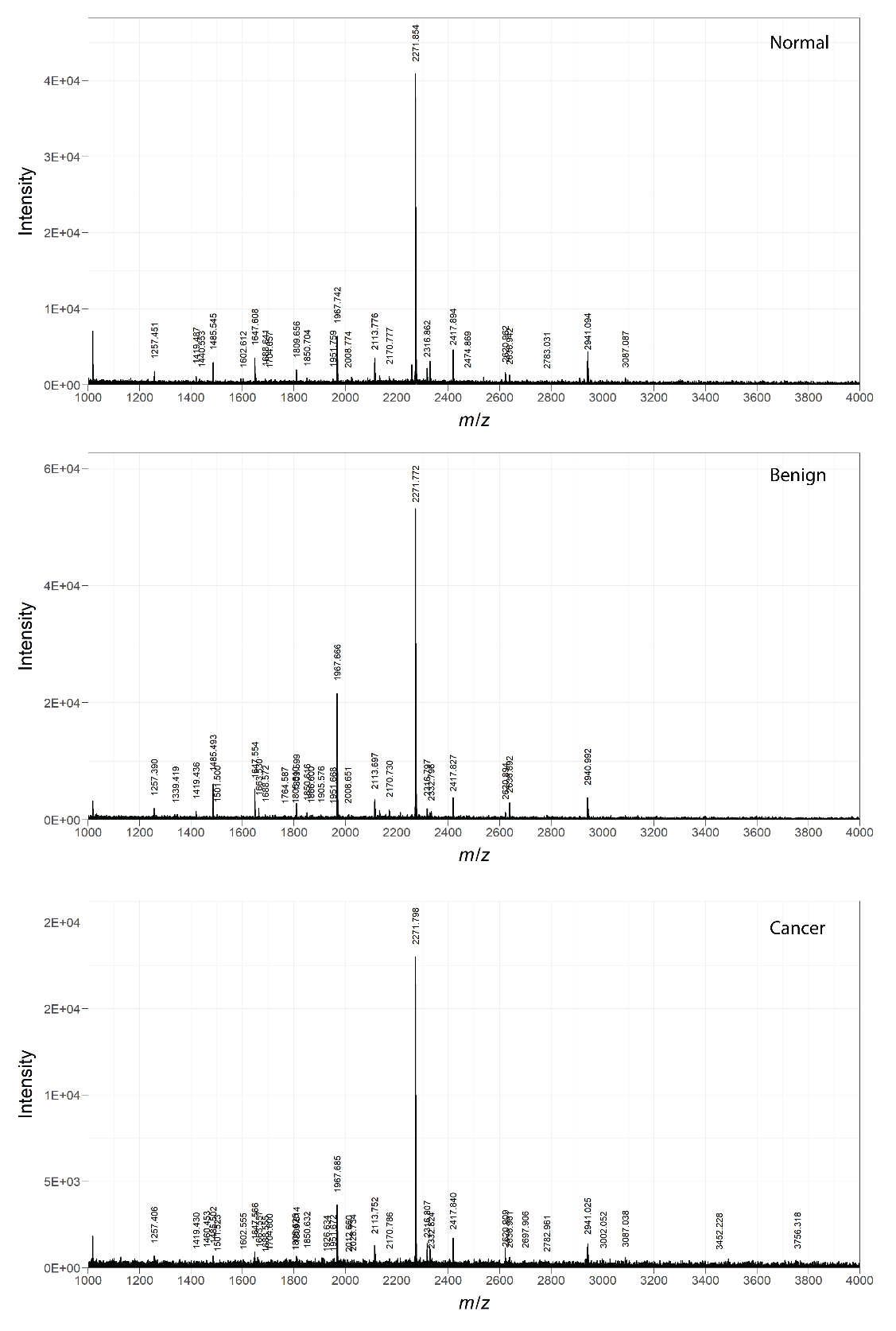


**Fig S2** Representative MALDI-MS spectra of serum *N*-glycans in the comparison between controls, benign neoplasm and ovarian cancer patients.


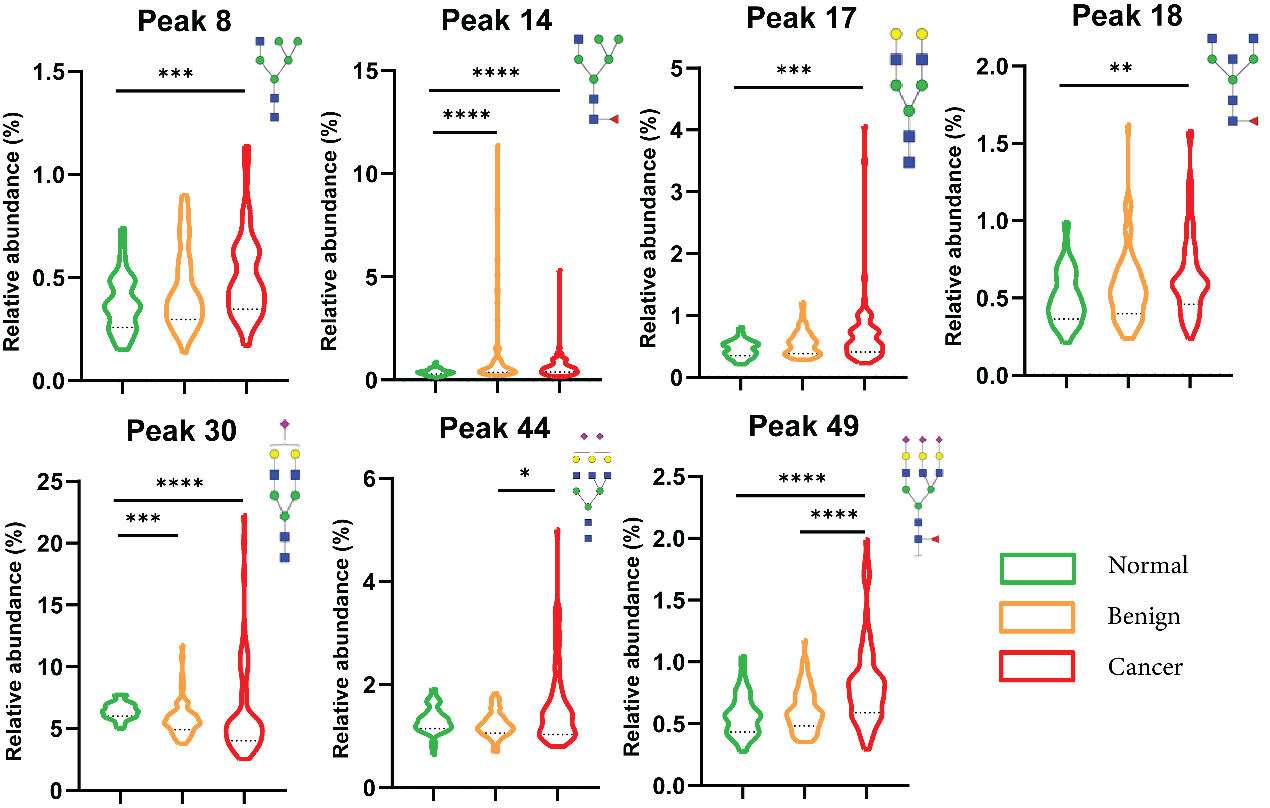


**Fig S3** Violin plots of the individual *N*-glycans in the comparison between normal and ovarian disease patients. Blue square, N-acetylglucosamine (N); green circle, mannose (H); yellow circle, galactose (H); purple diamond, sialic acid (S); red triangle, fucose (F). Significance is represented as not significant (n.s) *P* > 0.05, **P* ≤ 0.05, ***P* ≤ 0.01, ****P* ≤ 0.005, *****P* ≤ 0.001, Kruskal-Walli’s test with post Dunn’s multiple correction.


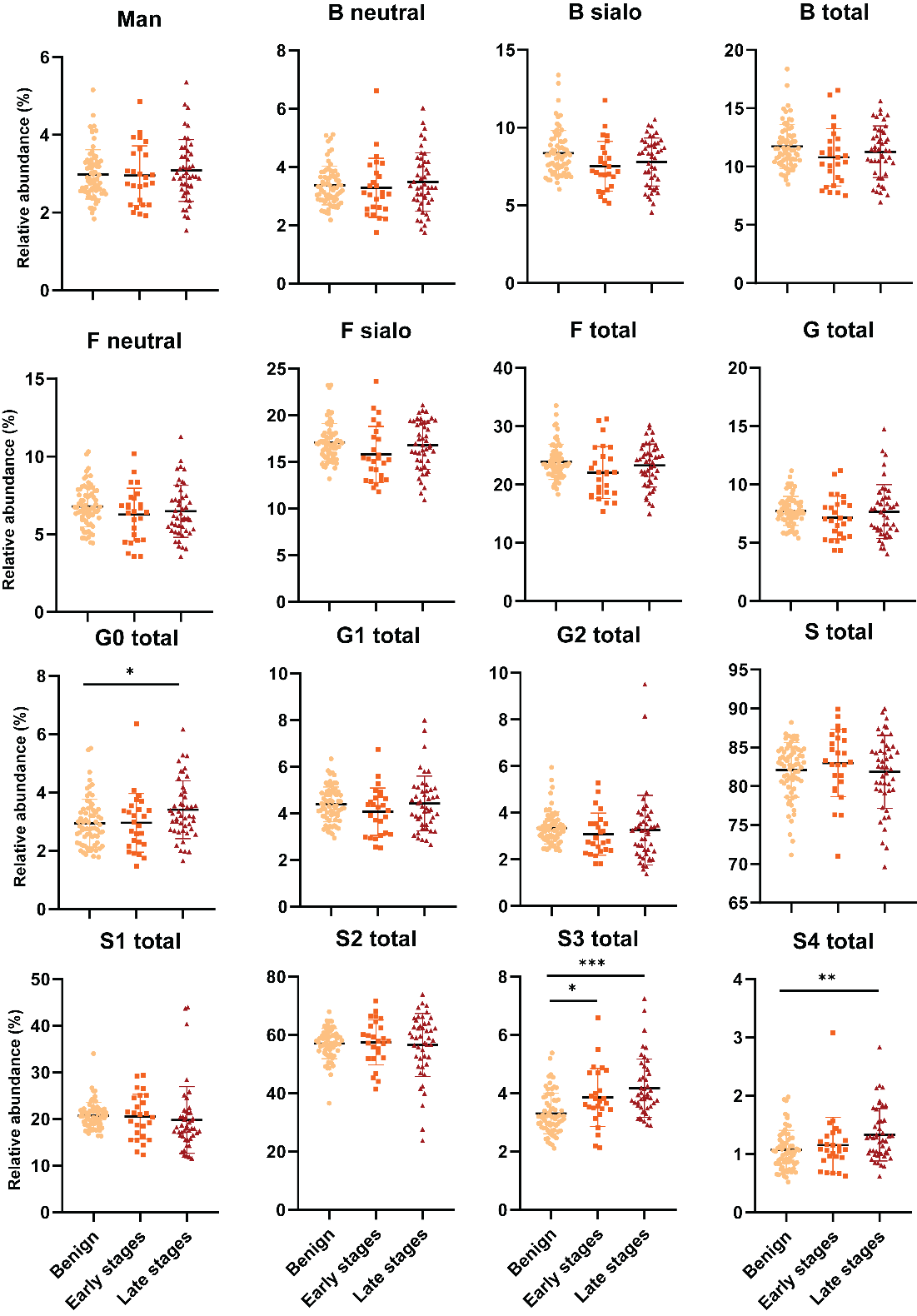


**Fig S4** Differentially expressed serum *N*-glycan derived traits in the comparison between benign and different stages of OC patients. Significance is represented as not significant (n.s) *P* > 0.05, **P* ≤ 0.05, ***P* ≤ 0.01, ****P* ≤ 0.005, *****P* ≤ 0.001, ANOVA test with post Bonferroni multiple correction.


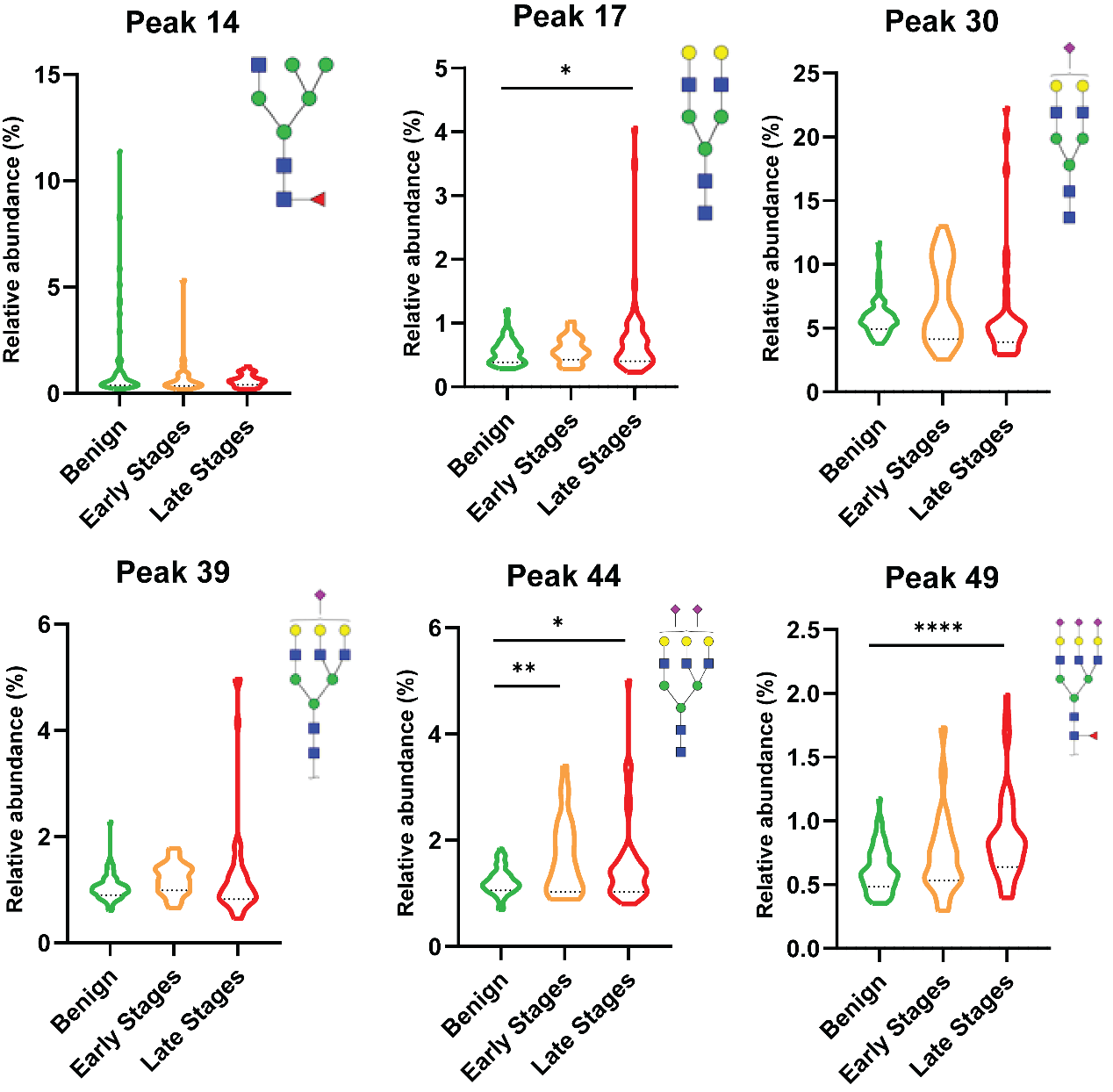


**Fig S5** Violin plots of the individual N-glycans in the comparison between benign and different stages of OC patients. Significance is represented as not significant (n.s) *P* > 0.05, **P* ≤ 0.05, ***P* ≤ 0.01, ****P* ≤ 0.005, *****P* ≤ 0.001, Kruskal-Walli’s test with post Dunn’s multiple correction.


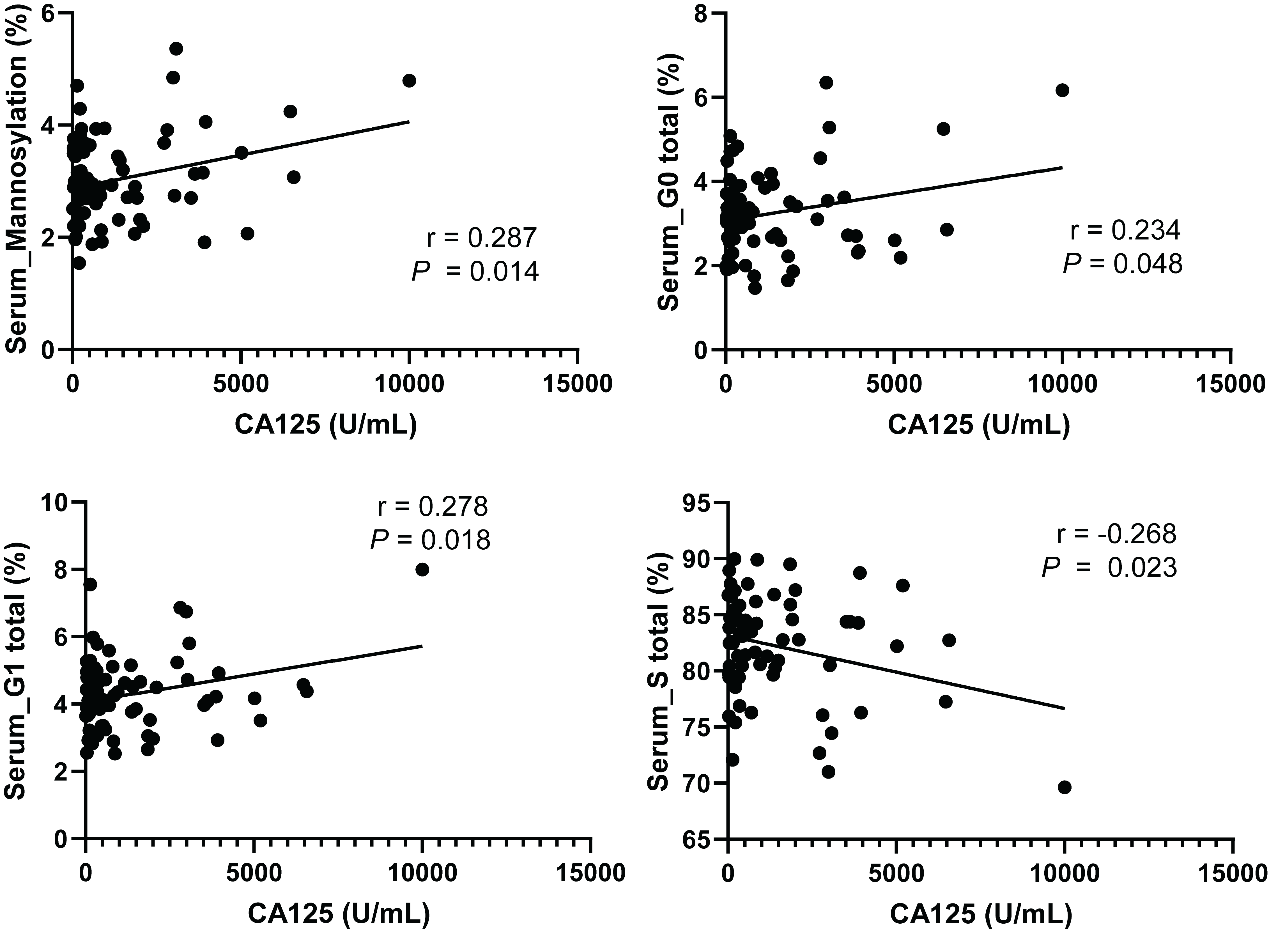


**Figure S6** Pearson correlation between serum glycosylation and CA 125. r, correlation coefficient.
